# Towards a symbiotic relationship between big data, artificial intelligence, and hospital pharmacy

**DOI:** 10.1186/s40545-020-00276-6

**Published:** 2020-11-09

**Authors:** Carlos Del Rio-Bermudez, Ignacio H. Medrano, Laura Yebes, Jose Luis Poveda

**Affiliations:** 1 Savana Medica, Madrid, Spain; 2 Pharmacy Department, Drug Clinical Area, University and Polytechnic Hospital La Fe, Avda. Fernando Abril Martorell 106, 46026 Valencia, Spain

**Keywords:** Natural language processing, Electronic health records, Machine learning, Pharmacovigilance

## Abstract

The digitalization of health and medicine and the growing availability of electronic health records (EHRs) has encouraged healthcare professionals and clinical researchers to adopt cutting-edge methodologies in the realms of artificial intelligence (AI) and big data analytics to exploit existing large medical databases. In Hospital and Health System pharmacies, the application of natural language processing (NLP) and machine learning to access and analyze the unstructured, free-text information captured in millions of EHRs (e.g., medication safety, patients’ medication history, adverse drug reactions, interactions, medication errors, therapeutic outcomes, and pharmacokinetic consultations) may become an essential tool to improve patient care and perform real-time evaluations of the efficacy, safety, and comparative effectiveness of available drugs. This approach has an enormous potential to support share-risk agreements and guide decision-making in pharmacy and therapeutics (P&T) Committees.

## Introduction

Healthcare settings gather and store large digital sets of patient data resulting from routine medical examinations, prescriptions, genome sequencing, laboratory testing, and administrative claims [1, 2]; most of this information ends up reflected in patients’ electronic health records (EHRs) [1]. In the context of Hospital and Health System Pharmacy, over 75% of hospital pharmacists in the US alone use data-mining functionalities to regularly document and collect patient-centered data [3]. These include key information regarding medication safety, medication history, and therapeutic outcomes [4, 5].

Due to the vast and heterogeneous nature of the digital data sets currently generated in health care settings, classical computing and research methods are no longer suited to handle and analyze this information. In response to these limitations, recent advancements in the realms of artificial intelligence (AI), most notably machine learning and natural language processing (NLP) have markedly improved the extraction, organization, and analysis of large amounts of clinical data regardless of their structure and linguistic complexity. However, despite these contributions, extra efforts are needed towards implementing these methods in the context of Hospital Pharmacy.

The analysis of massive amounts of data generated in Hospital Pharmacy settings [3] has enormous potential to unlock novel research questions and insights into patient management and drug safety. Furthermore, the adoption of these techniques may boost the visibility and recognition of Hospital Pharmacy, which is often demanded by hospital pharmacists and related professionals [6].

## Big data in hospital pharmacy settings

**Fig. 1:**
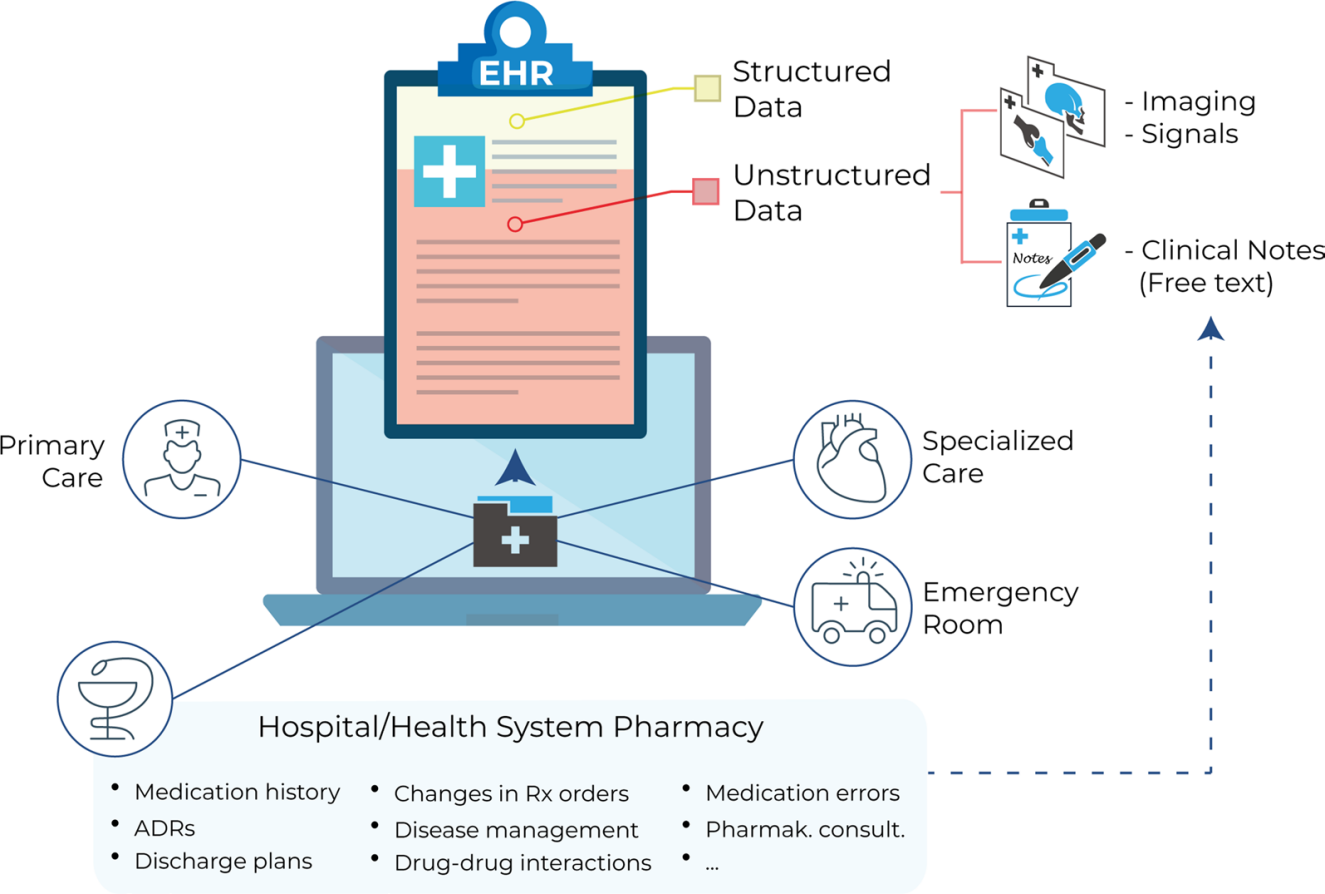
**Electronic health records (EHRs) contain a large amount of patient-centered clinical data**. EHRs are generated and stored in virtually all healthcare departments, including primary care and specialized care settings, emergency rooms, and hospital pharmacies. Most of the information captured in EHRs is unstructured (red shade), including imaging results/signals and free-text narratives jotted down by health professionals (clinical notes). Hospital pharmacists’ documentation of their interventions generate a vast amount of important clinical data, including patients’ medication history, adverse drug reactions (ADRs), discharge plans, changes in prescription (Rx) orders, disease management, drug–drug and other interactions, and pharmacokinetic consultations

The term ‘big data’ is commonly used to refer to datasets that are too large and heterogeneous to be stored and analyzed using traditional research methods [2, 7]. In Hospital and Health System pharmacies, big datasets result from the documentation of pharmacists’ interventions, medication reconciliation, and patient monitoring. As a direct result of these activities, patients’ EHRs contain large amounts of valuable information, including medication history, adverse drug reactions, interactions, medication errors, and pharmacokinetic consultations [3, 5] (Fig. 1).

### A golden ticket to real-time, real-world evidence (RWE)

To understand the full potential of the data captured in EHRs in assisting clinical research and practice, we must consider the following:

- *EHRs are widely and growingly available.* By 2015, more than 94% of hospitals in the US already had a certified EHR system [8]; this trend was closely followed by most developed countries. EHRs are generated and stored in virtually all healthcare departments, including primary care and specialized care settings, emergency rooms, and hospital pharmacies (Fig. 1).
- *The information captured in EHRs is rich and heterogeneous*. EHRs contain information regarding prescriptions, treatment outcomes, sociodemographic characteristics, previous comorbidities, test results, differential diagnosis, procedures, genetic background, signs and symptoms, family medical history, and lifestyle habits [1].
- *EHRs contain longitudinal data at the single-patient level*. As records are updated over time, they are suitable to address clinical questions that require regular patient follow-up and to predict outcomes at different stages of the patient’s journey [9].
- *The information captured in EHRs is scalable*. Regardless of the original structure and format, data contained in EHRs can be aggregated across patients and healthcare sites, and can be integrated with other valuable sources of health-related information such as genetic databanks, nutrition or health apps, census, and social media [10].

## Unlocking the full potential of clinical digital data: free-text narratives in EHRs

Routinely acquired medical data can be classified into structured and unstructured data. Whereas structured data refer to information that is stored in a consistent, organized manner and is typically reported using standard units and ranges (e.g., laboratory results, vital signs, ICD-based categorical diagnosis), unstructured data are devoid of a clear organization and precision (e.g., imaging results, clinical notes) [1]. Crucially, the majority of available clinical data in EHRs are unstructured [11] (Fig. 1).

The free-text narratives jotted down by health professionals (including physicians, nurses, and hospital pharmacists [3]) in EHRs reflect current clinical practices and provide a window into real-time, real-world clinical data. However, the complexity of the free text poses a significant methodological bottleneck to access, organize, and analyze written language with big data analytics.

### Accessing the free-text information in EHRs: the role of AI and NLP

The extraction of written text from EHRs is achieved through a combination of NLP and machine learning techniques. NLP is a field that borrows concepts and techniques from linguistics, computer science, and engineering to process naturally occurring language (i.e., speech or text), whereas machine learning models enable computers to extract patterns in datasets and draw conclusions on their own. Deep learning classification methods, which feed and learn from large amounts of data in EHRs, are used to teach the system to describe medical entities in terms of negative, speculative, or affirmative clinical statements. The extracted and processed information is then structured with artificial neural networks. Finally, analytical tools such as random forests, decision trees, and logistic regression enable the construction and visualization of predictive models derived from EHR data.

Extracting clinical information from free text is certainly challenging [7]. The main difficulties revolve around incorporating essential features of language, including temporal relationships, context, homonym use, and acronyms. A recent systematic review on the use of NLP to extract clinical information also pointed out other important technological gaps regarding concept understanding, causal inferences, and external validation of NLP-extracted data with annotated clinical corpora [12]. Despite these limitations, NLP is a cost-effective clinical tool; it has been estimated that 1 h of NLP system development saves at least 20 h of manual reviewing of medical records, with optimal sensitivity and specificity [13].

### EHRs and big data advance healthcare delivery

The effective exploitation of big data is thought to advance healthcare delivery by promoting the following actions [7, 11].

#### Generation and dissemination of data-driven medical knowledge in a timely fashion

The costs and time associated with manual data collection largely surpass those associated with the use of automatized tools. The combination of machine learning and NLP to explore EHRs has offered novel descriptive and predictive insights into clinical populations [9], patient management [14], and pharmacovigilance [15], and shows great promise for the generation of computerized clinical decision support (CDS) [16].

#### Personalized care

By integrating patients’ ‘-omics’ data (i.e., genomics, proteomics, microbiomics) with the information captured in EHRs, the Electronic Medical Records and Genomics (eMERGE) Network [17] has already identified unknown associations between patients’ genetic information and the clinical information in their EHRs in diverse therapeutic areas including ophthalmological and cardiovascular diseases.

#### Healthcare management and optimization of resource use

Clinical information in EHRs can be exploited to perform real-time predictive analyses to optimize resource use and management in terms of cost–benefit analysis. Relevant predictive outcomes achieved via analysis of EHR data include identification of risk factors associated to high-cost patients, readmissions, triage, and decompensation [7].

### Improving the state of the art in EHR studies

**Fig. 2:**
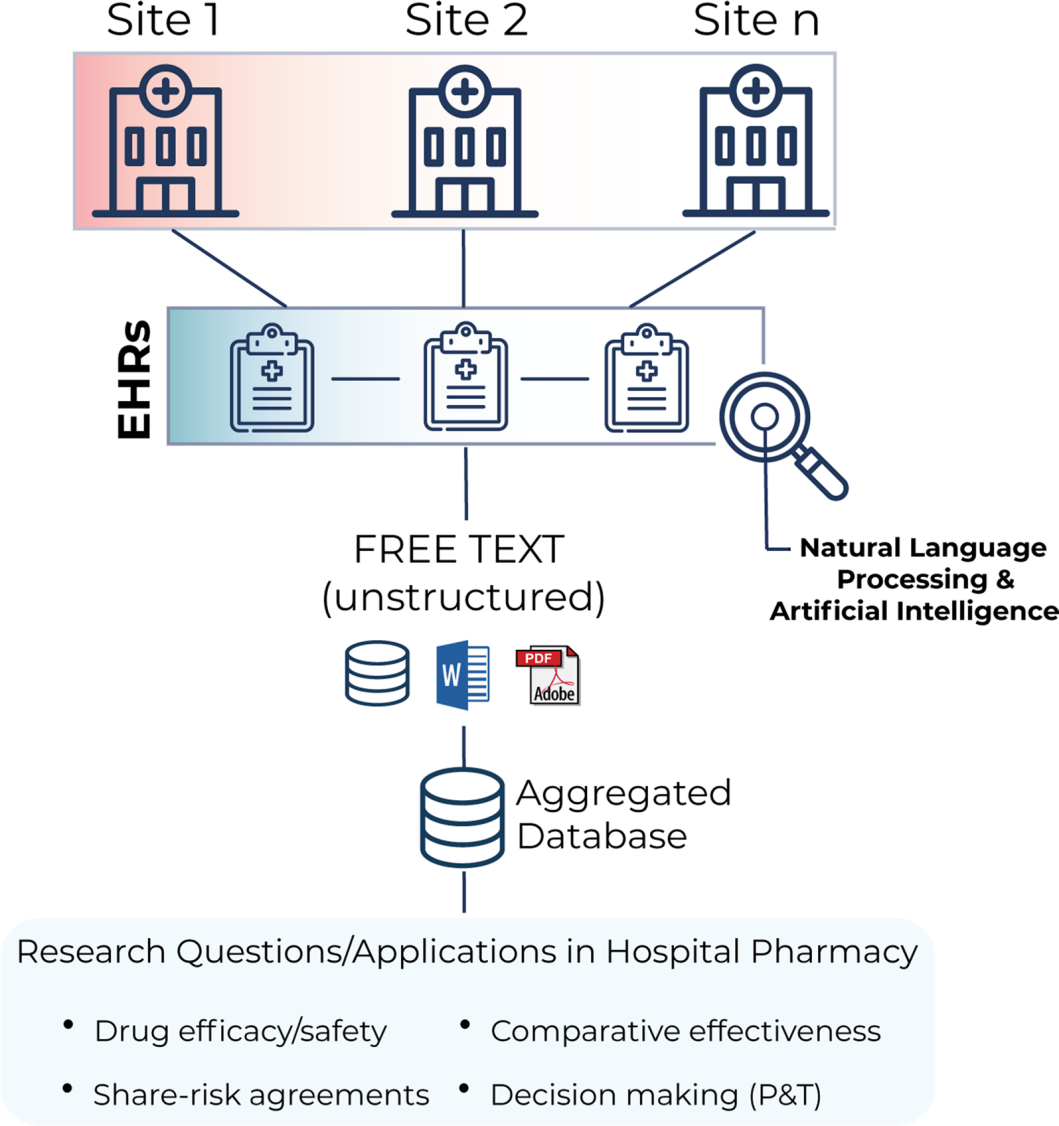
**Using natural language processing (NLP) and artificial intelligence (AI) to perform research studies with EHRs**. Adopting a multicenter approach, the free-text unstructured information (e.g., clinical notes in any digital format) in millions of de-identified EHRs can be organized in aggregated databases using NLP and AI. These tools are currently being used to answer important clinical questions in Hospital Pharmacy settings in real time, such as drug efficacy/safety and comparative effectiveness; these can be used to guide share-risk agreements and decision-making in pharmacy and therapeutics (P&T) committees

To move the field forward, we believe that the following three aspects should be considered in NLP research using EHRs. First, these studies always benefit from a multicentric, multilanguage methodology; unlike single-center studies, this approach enables access to even larger datasets (in turn generating more accurate predictive models), inclusion of more diverse study populations, and the possibility of comparing results across centers and regions. Second, the output of a clinical NLP system should always validated against a corpus of expert-reviewed clinical notes in terms of sensitivity and recall of extracted medical concepts [14]. Finally, researches must always guarantee the confidentiality and security of the data, in compliance with hospital ethics committees, national and international regulations, and pharmaceutical industry policies. Following these recommendations, the use of available research tools such as the EHRead^®^ technology now allows researchers to rapidly answer clinical questions in real time using patient-centered data [14, 18, 19]. A summary of this methodological approach is depicted in Fig. 2.

## Applications and challenges in the future of hospital pharmacy

Although system pharmacies may have lagged behind in their use of AI, the application of NLP and machine learning to extract and analyze unstructured information in EHRs has already provided valuable insights into pharmacovigilance, including identification of drug-related adverse events that were previously unknown, identification of discrepancies in patients’ medications and errors in prescriptions based on comorbidities and risk factors, and adherence to treatment monitoring [15].

EHRs also contain relevant drug cost data that can be used to evaluate treatment options and palliate the financial burden of patients [2]. The realization that increasing out-of-pocket expenditures for patients worsens treatment adherence and causes larger downstream costs has ignited a big push for disclosing drug costs on EHRs [20]. With this information on EHRs, large-scale analyses can be conducted to allow prescribers to offer the most cost-effective medications to their patients.

Altogether, the application of NLP and machine learning to analyze patients’ EHRs may become an essential tool to perform real-time evaluations of the efficacy, safety, and comparative effectiveness of available drugs (including those currently in post-marketing surveillance). This may in turn facilitate share-risk agreements and assist decision-making in pharmacy and therapeutics (P&T) Committees [5] (Fig. 2).

### Current challenges

#### What to document, how, and when

As integral elements of the Health System, hospital pharmacists are ethically obliged to document the care they provide in patients’ health records. However, there are some discrepancies and controversies around (a) standard guidelines for the recording and documentation of hospital pharmacists’ activities, (b) reporting ‘near miss’ and other interventions regarding potential risks for adverse events that have been successfully intercepted, and (c) lack of standardization of EHR data across hospital sites [4].

#### Privacy and security

A recurrent concern with the secondary use of EHRs and is data privacy and how it affects data sharing. To this end, tools and procedures have been created to de-identify (i.e., make anonymous and untraceable to single patients) EHRs. Once EHRs are de-identified, they can be used for research and clinical purposes since they are no longer subject to country- and region-specific privacy regulations for identifiable patient information. However, as the potential for data aggregation and linkage across data sources grows exponentially, doubts loom large for de-identification procedures and their actual efficiency.

#### Interoperability: data availability and data sharing

Though often based on misconceptions around data privacy and security, existing concerns by policy makers, hospital managers, and local regulatory agencies have limited data availability and sharing among professionals and clinical researchers. The application of big data analytics in healthcare is an unescapable reality in need of solid regulations that facilitate data availability and sharing. Precisely, big data relies on the principle of interoperability, which translates into the ability to exchange data across organizational boundaries in a timely fashion.

## Concluding remarks

The clinical data captured in EHRs provide RWE and can be aggregated on a large scale to describe patient populations and offer predictive insights into disease prognosis, treatment responses, and resource use in healthcare settings. Using available tools in the fields of machine learning and NLP, clinical pharmacists can exploit the vast amounts of clinical data collected in their daily practice.

Realizing that these large datasets are important resources to improve healthcare rather than mere byproducts of its delivery is the first step to unlock the full potential of the data generated in hospital pharmacies, improve the needed visibility of services provided and recognition of Hospital Pharmacy professionals [6], and above all, promote excellence in patient care.
